# Supplementary material for: Hot spot prediction in protein-protein interactions by an ensemble system
Source: BMC Syst Biol. 2018 Dec 31;12(Suppl 9):132. doi: 10.1186/s12918-018-0665-8 (PMC6311905; doi:10.1186/s12918-018-0665-8)
Supplement: Supplementary file 2 — All relevant MATLAB code. A simple Matlab implement of our predictor. (ZIP 19,742 kb) [file 12918_2018_665_MOESM2_ESM.zip › Additional file 2/Read ASA/ASA website/BID/1G3IA.html]

 NetSurfP - Protein Surface Accessibility and Secondary Structure Predictions

  

|  |  |  |
| --- | --- | --- |
|  |  | NetSurfP - Protein Surface Accessibility and Secondary Structure Predictions  Technical University of Denmark |

  


---

```
# For publication of results, please cite:
# A generic method for assignment of reliability scores applied to solvent accessibility predictions.
# Bent Petersen, Thomas Nordahl Petersen, Pernille Andersen, Morten Nielsen and Claus Lundegaard
# BMC Structural Biology 2009, 9:51 doi:10.1186/1472-6807-9-51
#
# Column 1: Class assignment - B for buried or E for Exposed - Threshold: 25% exposure, but not based on RSA
# Column 2: Amino acid
# Column 3: Sequence name
# Column 4: Amino acid number
# Column 5: Relative Surface Accessibility - RSA
# Column 6: Absolute Surface Accessibility
# Column 7: Z-fit score for RSA prediction
# Column 8: Probability for Alpha-Helix
# Column 9: Probability for Beta-strand
# Column 10: Probability for Coil
E M  1G3IA                  1    0.716 143.192  -2.118   0.003   0.003   0.994
E S  1G3IA                  2    0.789  92.447   0.179   0.018   0.019   0.964
E E  1G3IA                  3    0.650 113.555   0.492   0.018   0.019   0.964
B M  1G3IA                  4    0.179  35.918  -0.450   0.058   0.017   0.925
E T  1G3IA                  5    0.464  64.301   1.617   0.053   0.005   0.942
B P  1G3IA                  6    0.160  22.761  -0.331   0.600   0.003   0.397
E R  1G3IA                  7    0.557 127.530   1.507   0.717   0.014   0.269
E E  1G3IA                  8    0.493  86.057   1.117   0.717   0.014   0.269
B I  1G3IA                  9    0.069  12.709   0.837   0.717   0.014   0.269
E V  1G3IA                 10    0.253  38.917   1.189   0.751   0.050   0.199
E S  1G3IA                 11    0.599  70.168   1.652   0.751   0.050   0.199
E E  1G3IA                 12    0.324  56.603   1.513   0.751   0.050   0.199
B L  1G3IA                 13    0.061  11.224   1.113   0.622   0.015   0.363
B D  1G3IA                 14    0.254  36.573   0.403   0.622   0.015   0.363
E Q  1G3IA                 15    0.697 124.502   1.161   0.622   0.015   0.363
B H  1G3IA                 16    0.271  49.277   0.386   0.717   0.014   0.269
B I  1G3IA                 17    0.047   8.658   0.146   0.660   0.049   0.291
B I  1G3IA                 18    0.181  33.503   0.142   0.561   0.047   0.393
E G  1G3IA                 19    0.309  24.295   0.342   0.257   0.016   0.727
B Q  1G3IA                 20    0.149  26.576   1.140   0.717   0.014   0.269
E A  1G3IA                 21    0.563  62.054   0.906   0.923   0.002   0.076
E D  1G3IA                 22    0.495  71.402   1.299   0.970   0.001   0.030
B A  1G3IA                 23    0.032   3.515   0.362   0.970   0.001   0.030
B K  1G3IA                 24    0.183  37.705   1.568   0.988   0.000   0.012
E R  1G3IA                 25    0.497 113.836   1.772   0.988   0.000   0.012
E A  1G3IA                 26    0.264  29.071   2.097   0.988   0.000   0.012
B V  1G3IA                 27    0.013   2.013   1.836   0.988   0.000   0.012
B A  1G3IA                 28    0.207  22.866   1.794   0.988   0.000   0.012
E I  1G3IA                 29    0.301  55.667   1.249   0.970   0.001   0.030
B A  1G3IA                 30    0.046   5.058   1.136   0.975   0.003   0.022
B L  1G3IA                 31    0.024   4.431   0.923   0.938   0.007   0.055
E R  1G3IA                 32    0.357  81.707   0.842   0.802   0.014   0.185
B N  1G3IA                 33    0.209  30.598   0.304   0.406   0.004   0.590
B R  1G3IA                 34    0.189  43.235  -0.516   0.246   0.004   0.750
B W  1G3IA                 35    0.211  50.818  -0.439   0.246   0.004   0.750
E R  1G3IA                 36    0.447 102.386   0.159   0.246   0.004   0.750
E R  1G3IA                 37    0.351  80.425  -0.496   0.176   0.004   0.820
E M  1G3IA                 38    0.431  86.243  -0.870   0.321   0.003   0.675
E Q  1G3IA                 39    0.603 107.767  -0.437   0.430   0.016   0.555
E L  1G3IA                 40    0.288  52.788  -0.462   0.522   0.016   0.462
E Q  1G3IA                 41    0.295  52.687  -0.740   0.522   0.016   0.462
E E  1G3IA                 42    0.600 104.837  -0.927   0.622   0.015   0.363
E P  1G3IA                 43    0.691  98.053  -0.083   0.622   0.015   0.363
E L  1G3IA                 44    0.336  61.467  -0.886   0.430   0.016   0.555
E R  1G3IA                 45    0.607 139.095  -1.668   0.115   0.016   0.868
E H  1G3IA                 46    0.388  70.632  -1.717   0.053   0.043   0.903
E E  1G3IA                 47    0.562  98.216  -0.713   0.056   0.142   0.802
B V  1G3IA                 48    0.231  35.474  -0.160   0.064   0.216   0.721
E T  1G3IA                 49    0.311  43.108  -0.010   0.018   0.088   0.893
B P  1G3IA                 50    0.260  36.922  -0.477   0.018   0.047   0.935
E K  1G3IA                 51    0.286  58.892   0.009   0.018   0.047   0.935
B N  1G3IA                 52    0.066   9.662  -0.726   0.004   0.197   0.799
B I  1G3IA                 53    0.021   3.903   0.920   0.003   0.718   0.279
B L  1G3IA                 54    0.024   4.394   0.343   0.001   0.900   0.099
B M  1G3IA                 55    0.022   4.362   0.512   0.001   0.900   0.099
B I  1G3IA                 56    0.039   7.178  -0.365   0.003   0.718   0.279
B G  1G3IA                 57    0.043   3.368  -0.385   0.004   0.197   0.799
B P  1G3IA                 58    0.093  13.253   0.016   0.005   0.015   0.979
B T  1G3IA                 59    0.149  20.736  -1.138   0.003   0.003   0.994
B G  1G3IA                 60    0.207  16.291  -2.277   0.016   0.005   0.979
B V  1G3IA                 61    0.087  13.449  -0.829   0.109   0.005   0.886
B G  1G3IA                 62    0.056   4.423  -1.100   0.321   0.003   0.675
B K  1G3IA                 63    0.181  37.293   0.223   0.923   0.002   0.076
B T  1G3IA                 64    0.178  24.744  -0.271   0.923   0.002   0.076
B E  1G3IA                 65    0.164  28.738   0.436   0.970   0.001   0.030
B I  1G3IA                 66    0.041   7.677   0.184   0.970   0.001   0.030
B A  1G3IA                 67    0.040   4.408  -0.255   0.988   0.000   0.012
E R  1G3IA                 68    0.245  56.036   1.328   0.970   0.001   0.030
B R  1G3IA                 69    0.095  21.640   0.534   0.970   0.001   0.030
B L  1G3IA                 70    0.047   8.679   0.679   0.970   0.001   0.030
B A  1G3IA                 71    0.046   5.069   0.202   0.923   0.002   0.076
E K  1G3IA                 72    0.424  87.217   0.822   0.782   0.003   0.216
B L  1G3IA                 73    0.210  38.414   0.044   0.717   0.014   0.269
B A  1G3IA                 74    0.069   7.615  -0.519   0.430   0.016   0.555
E N  1G3IA                 75    0.579  84.824   0.164   0.018   0.019   0.964
B A  1G3IA                 76    0.041   4.474   0.705   0.018   0.047   0.935
B P  1G3IA                 77    0.236  33.503  -0.011   0.005   0.262   0.733
B F  1G3IA                 78    0.054  10.858   0.679   0.001   0.900   0.099
B I  1G3IA                 79    0.077  14.245   0.358   0.001   0.959   0.040
B K  1G3IA                 80    0.179  36.861   0.477   0.001   0.959   0.040
B V  1G3IA                 81    0.052   7.916  -0.287   0.001   0.900   0.099
E E  1G3IA                 82    0.384  67.137   0.336   0.003   0.718   0.279
B A  1G3IA                 83    0.070   7.736  -0.442   0.020   0.205   0.775
E T  1G3IA                 84    0.343  47.630  -0.095   0.052   0.084   0.864
E K  1G3IA                 85    0.473  97.276   0.519   0.125   0.227   0.648
B F  1G3IA                 86    0.154  30.888   0.092   0.135   0.317   0.548
B T  1G3IA                 87    0.237  32.914  -0.273   0.135   0.317   0.548
E E  1G3IA                 88    0.403  70.387  -1.069   0.231   0.330   0.439
B V  1G3IA                 89    0.289  44.450  -0.432   0.125   0.227   0.648
E G  1G3IA                 90    0.368  28.946  -1.293   0.118   0.150   0.732
B Y  1G3IA                 91    0.269  57.400  -0.165   0.199   0.152   0.649
B V  1G3IA                 92    0.228  35.090  -0.674   0.191   0.086   0.723
B G  1G3IA                 93    0.152  11.978  -1.121   0.622   0.015   0.363
E K  1G3IA                 94    0.594 122.186   0.691   0.717   0.014   0.269
E E  1G3IA                 95    0.337  58.856   1.356   0.802   0.014   0.185
B V  1G3IA                 96    0.032   4.965   0.352   0.970   0.001   0.030
E D  1G3IA                 97    0.307  44.282   1.234   0.970   0.001   0.030
E S  1G3IA                 98    0.499  58.448   1.662   0.970   0.001   0.030
B I  1G3IA                 99    0.044   8.048   1.103   0.970   0.001   0.030
B I  1G3IA                100    0.035   6.438   0.294   0.970   0.001   0.030
E R  1G3IA                101    0.460 105.248   2.189   0.970   0.001   0.030
E D  1G3IA                102    0.425  61.300   2.173   0.970   0.001   0.030
B L  1G3IA                103    0.018   3.241   1.274   0.970   0.001   0.030
E T  1G3IA                104    0.340  47.213   1.361   0.923   0.002   0.076
E D  1G3IA                105    0.684  98.564   1.106   0.858   0.002   0.139
E S  1G3IA                106    0.381  44.700   0.316   0.600   0.003   0.397
B A  1G3IA                107    0.048   5.290  -0.108   0.109   0.005   0.886
E M  1G3IA                108    0.426  85.283   0.317   0.016   0.005   0.979
B K  1G3IA                109    0.091  18.698  -0.149   0.018   0.088   0.893
B L  1G3IA                110    0.067  12.268   0.928   0.022   0.552   0.426
B V  1G3IA                111    0.060   9.299   1.027   0.072   0.855   0.073
B R  1G3IA                112    0.100  22.877   1.167   0.085   0.775   0.140
B Q  1G3IA                113    0.136  24.236   1.103   0.487   0.370   0.144
E Q  1G3IA                114    0.381  68.029   0.249   0.725   0.163   0.112
B E  1G3IA                115    0.185  32.267   0.213   0.831   0.044   0.125
B I  1G3IA                116    0.102  18.962   0.734   0.879   0.010   0.111
E A  1G3IA                117    0.375  41.292   0.817   0.802   0.014   0.185
E K  1G3IA                118    0.462  94.931   1.268   0.802   0.014   0.185
B N  1G3IA                119    0.128  18.798   0.280   0.717   0.014   0.269
B R  1G3IA                120    0.219  50.082   0.584   0.694   0.003   0.303
E A  1G3IA                121    0.631  69.536   1.254   0.694   0.003   0.303
E R  1G3IA                122    0.530 121.484   1.052   0.717   0.014   0.269
B A  1G3IA                123    0.215  23.715  -0.440   0.622   0.015   0.363
E E  1G3IA                124    0.598 104.436  -0.286   0.522   0.016   0.462
E D  1G3IA                125    0.607  87.512   0.077   0.522   0.016   0.462
E V  1G3IA                126    0.394  60.496  -0.485   0.622   0.015   0.363
E A  1G3IA                127    0.321  35.341  -1.048   0.600   0.003   0.397
E E  1G3IA                128    0.348  60.831  -0.860   0.782   0.003   0.216
E E  1G3IA                129    0.509  88.852  -0.053   0.923   0.002   0.076
E R  1G3IA                130    0.377  86.425   0.718   0.938   0.007   0.055
B I  1G3IA                131    0.092  17.094   0.931   0.938   0.007   0.055
B L  1G3IA                132    0.097  17.816   0.779   0.938   0.007   0.055
E D  1G3IA                133    0.492  70.883   0.391   0.879   0.010   0.111
E A  1G3IA                134    0.316  34.834   0.972   0.802   0.014   0.185
B L  1G3IA                135    0.118  21.624   0.180   0.717   0.014   0.269
B L  1G3IA                136    0.119  21.697   0.778   0.339   0.016   0.645
E P  1G3IA                137    0.529  75.079   0.546   0.181   0.016   0.803
E P  1G3IA                138    0.377  53.539  -0.286   0.181   0.016   0.803
B A  1G3IA                139    0.281  30.911  -0.726   0.181   0.016   0.803
E K  1G3IA                140    0.682 140.205  -0.610   0.115   0.016   0.868
E N  1G3IA                141    0.580  84.912  -1.803   0.115   0.016   0.868
E Q  1G3IA                142    0.311  55.580  -1.819   0.058   0.017   0.925
E W  1G3IA                143    0.598 143.891  -1.518   0.115   0.016   0.868
E G  1G3IA                144    0.564  44.371  -1.578   0.113   0.043   0.844
E E  1G3IA                145    0.577 100.784  -1.153   0.113   0.087   0.800
E V  1G3IA                146    0.339  52.043  -1.216   0.118   0.150   0.732
E E  1G3IA                147    0.538  94.006  -0.683   0.125   0.227   0.648
E N  1G3IA                148    0.339  49.615  -0.410   0.216   0.235   0.548
E H  1G3IA                149    0.427  77.617  -0.144   0.199   0.152   0.649
E D  1G3IA                150    0.526  75.825  -1.177   0.191   0.086   0.723
E S  1G3IA                151    0.406  47.618  -1.584   0.184   0.043   0.773
E H  1G3IA                152    0.413  75.125  -0.669   0.184   0.043   0.773
E S  1G3IA                153    0.576  67.531  -0.386   0.257   0.016   0.727
E S  1G3IA                154    0.387  45.310   0.332   0.181   0.016   0.803
E T  1G3IA                155    0.326  45.258   0.435   0.339   0.016   0.645
B R  1G3IA                156    0.254  58.258   0.703   0.858   0.002   0.139
E Q  1G3IA                157    0.622 111.089   0.368   0.858   0.002   0.139
E A  1G3IA                158    0.299  32.994   0.746   0.858   0.002   0.139
B F  1G3IA                159    0.034   6.844   0.107   0.923   0.002   0.076
E R  1G3IA                160    0.273  62.586   1.371   0.923   0.002   0.076
E K  1G3IA                161    0.617 126.917   1.221   0.923   0.002   0.076
B K  1G3IA                162    0.207  42.477   0.449   0.858   0.002   0.139
B L  1G3IA                163    0.107  19.555  -0.103   0.782   0.003   0.216
E R  1G3IA                164    0.374  85.600   0.117   0.600   0.003   0.397
E E  1G3IA                165    0.515  90.023   0.305   0.246   0.004   0.750
E G  1G3IA                166    0.382  30.079  -1.843   0.058   0.017   0.925
E Q  1G3IA                167    0.540  96.533  -1.190   0.018   0.019   0.964
E L  1G3IA                168    0.372  68.186  -1.347   0.058   0.017   0.925
E D  1G3IA                169    0.528  76.128  -1.085   0.058   0.017   0.925
E D  1G3IA                170    0.596  85.941  -1.234   0.018   0.019   0.964
E K  1G3IA                171    0.443  91.187  -0.610   0.018   0.088   0.893
E E  1G3IA                172    0.478  83.472  -0.650   0.004   0.514   0.481
B I  1G3IA                173    0.165  30.562  -0.310   0.002   0.816   0.182
E E  1G3IA                174    0.455  79.454  -0.409   0.011   0.918   0.071
B I  1G3IA                175    0.136  25.104  -0.061   0.018   0.846   0.136
E D  1G3IA                176    0.421  60.666  -1.505   0.023   0.655   0.322
B V  1G3IA                177    0.186  28.573  -0.603   0.022   0.359   0.619
E S  1G3IA                178    0.359  42.075  -0.692   0.020   0.205   0.775
E A  1G3IA                179    0.514  56.676  -0.962   0.064   0.216   0.721
E G  1G3IA                180    0.487  38.311  -1.787   0.064   0.216   0.721
E V  1G3IA                181    0.426  65.461  -1.398   0.064   0.216   0.721
E S  1G3IA                182    0.379  44.442  -1.668   0.125   0.227   0.648
E M  1G3IA                183    0.551 110.355  -1.624   0.118   0.150   0.732
E G  1G3IA                184    0.440  34.628  -2.214   0.118   0.150   0.732
E V  1G3IA                185    0.376  57.714  -1.805   0.125   0.227   0.648
E E  1G3IA                186    0.418  73.060  -1.919   0.066   0.296   0.638
B I  1G3IA                187    0.289  53.428  -2.620   0.064   0.216   0.721
E M  1G3IA                188    0.377  75.438  -2.477   0.064   0.216   0.721
E A  1G3IA                189    0.375  41.358  -1.949   0.056   0.142   0.802
E P  1G3IA                190    0.458  64.933  -2.005   0.113   0.087   0.800
E P  1G3IA                191    0.462  65.515  -2.358   0.184   0.043   0.773
E G  1G3IA                192    0.423  33.266  -1.552   0.257   0.016   0.727
B M  1G3IA                193    0.228  45.683  -1.419   0.622   0.015   0.363
E E  1G3IA                194    0.550  96.050  -0.295   0.717   0.014   0.269
E E  1G3IA                195    0.463  80.816   0.065   0.717   0.014   0.269
B M  1G3IA                196    0.116  23.212  -0.512   0.802   0.014   0.185
E T  1G3IA                197    0.289  40.070  -0.797   0.802   0.014   0.185
E N  1G3IA                198    0.556  81.457   0.019   0.802   0.014   0.185
E Q  1G3IA                199    0.396  70.761  -0.400   0.802   0.014   0.185
B L  1G3IA                200    0.083  15.289  -0.403   0.782   0.003   0.216
E Q  1G3IA                201    0.443  79.084  -0.583   0.782   0.003   0.216
E S  1G3IA                202    0.605  70.953  -0.699   0.782   0.003   0.216
E L  1G3IA                203    0.272  49.876  -0.058   0.694   0.003   0.303
B F  1G3IA                204    0.239  48.048  -1.533   0.622   0.015   0.363
E Q  1G3IA                205    0.503  89.800  -1.488   0.257   0.016   0.727
E N  1G3IA                206    0.582  85.190  -1.491   0.115   0.016   0.868
B L  1G3IA                207    0.235  43.083  -1.162   0.115   0.016   0.868
E G  1G3IA                208    0.373  29.347  -1.370   0.115   0.016   0.868
E S  1G3IA                209    0.337  39.461  -2.032   0.181   0.016   0.803
E D  1G3IA                210    0.571  82.339  -0.562   0.257   0.016   0.727
E K  1G3IA                211    0.383  78.886  -0.147   0.354   0.048   0.598
E T  1G3IA                212    0.399  55.300  -0.429   0.455   0.046   0.498
E K  1G3IA                213    0.612 125.868   0.208   0.455   0.046   0.498
E K  1G3IA                214    0.401  82.486   0.816   0.354   0.048   0.598
E R  1G3IA                215    0.532 121.874   0.610   0.278   0.093   0.628
E K  1G3IA                216    0.528 108.712   0.950   0.191   0.086   0.723
B M  1G3IA                217    0.133  26.573   1.194   0.191   0.086   0.723
E K  1G3IA                218    0.452  92.956   1.033   0.113   0.043   0.844
E I  1G3IA                219    0.297  55.019   0.511   0.879   0.010   0.111
E K  1G3IA                220    0.518 106.635   0.165   0.802   0.014   0.185
E D  1G3IA                221    0.497  71.546   0.856   0.782   0.003   0.216
B A  1G3IA                222    0.077   8.518  -0.271   0.782   0.003   0.216
E L  1G3IA                223    0.276  50.536   1.041   0.694   0.003   0.303
E K  1G3IA                224    0.544 111.901   0.817   0.622   0.015   0.363
B A  1G3IA                225    0.246  27.087   0.085   0.694   0.003   0.303
B L  1G3IA                226    0.077  14.135   0.279   0.802   0.014   0.185
B I  1G3IA                227    0.249  46.046   1.047   0.802   0.014   0.185
E D  1G3IA                228    0.494  71.157   0.467   0.923   0.002   0.076
E D  1G3IA                229    0.370  53.303   0.475   0.923   0.002   0.076
B E  1G3IA                230    0.185  32.232   0.299   0.923   0.002   0.076
B A  1G3IA                231    0.095  10.513  -0.179   0.923   0.002   0.076
E A  1G3IA                232    0.432  47.639   0.517   0.858   0.002   0.139
E K  1G3IA                233    0.400  82.342   1.172   0.782   0.003   0.216
B L  1G3IA                234    0.141  25.780   0.385   0.694   0.003   0.303
B I  1G3IA                235    0.212  39.202   0.047   0.502   0.002   0.495
E N  1G3IA                236    0.440  64.416   0.141   0.321   0.003   0.675
E P  1G3IA                237    0.334  47.380  -0.163   0.406   0.004   0.590
E E  1G3IA                238    0.563  98.286  -0.952   0.600   0.003   0.397
E E  1G3IA                239    0.534  93.290   0.282   0.622   0.015   0.363
B L  1G3IA                240    0.177  32.464   0.038   0.717   0.014   0.269
E K  1G3IA                241    0.341  70.205   0.593   0.802   0.014   0.185
E Q  1G3IA                242    0.551  98.319   0.447   0.923   0.002   0.076
E K  1G3IA                243    0.561 115.295   0.816   0.923   0.002   0.076
B A  1G3IA                244    0.185  20.387   0.299   0.858   0.002   0.139
B I  1G3IA                245    0.292  54.076   0.038   0.858   0.002   0.139
E D  1G3IA                246    0.509  73.347   0.244   0.782   0.003   0.216
E A  1G3IA                247    0.444  48.929   0.661   0.802   0.014   0.185
B V  1G3IA                248    0.221  33.906   0.103   0.802   0.014   0.185
E E  1G3IA                249    0.300  52.410  -0.614   0.717   0.014   0.269
E Q  1G3IA                250    0.537  95.926  -0.068   0.622   0.015   0.363
E N  1G3IA                251    0.471  68.881   0.307   0.354   0.048   0.598
E G  1G3IA                252    0.346  27.238  -0.534   0.113   0.087   0.800
B I  1G3IA                253    0.205  37.833   0.455   0.125   0.227   0.648
B V  1G3IA                254    0.269  41.330   0.073   0.159   0.516   0.325
B F  1G3IA                255    0.129  25.930   0.779   0.159   0.516   0.325
B I  1G3IA                256    0.170  31.376   0.239   0.148   0.418   0.435
E D  1G3IA                257    0.497  71.661   0.061   0.321   0.252   0.427
E E  1G3IA                258    0.450  78.702  -0.258   0.386   0.097   0.517
B I  1G3IA                259    0.120  22.108  -0.089   0.386   0.097   0.517
E D  1G3IA                260    0.432  62.237   0.272   0.268   0.043   0.689
E K  1G3IA                261    0.439  90.220   0.062   0.278   0.093   0.628
E I  1G3IA                262    0.403  74.629  -0.198   0.278   0.093   0.628
B C  1G3IA                263    0.271  38.048  -0.185   0.191   0.086   0.723
E K  1G3IA                264    0.426  87.649  -0.750   0.191   0.086   0.723
E K  1G3IA                265    0.514 105.771  -0.720   0.113   0.087   0.800
E G  1G3IA                266    0.557  43.828  -1.761   0.113   0.087   0.800
E E  1G3IA                267    0.663 115.913  -1.858   0.113   0.043   0.844
E Y  1G3IA                268    0.419  89.476  -2.043   0.053   0.043   0.903
E S  1G3IA                269    0.536  62.854  -1.296   0.058   0.017   0.925
E G  1G3IA                270    0.417  32.818  -1.472   0.113   0.043   0.844
E A  1G3IA                271    0.392  43.242  -0.563   0.113   0.087   0.800
B D  1G3IA                272    0.262  37.769  -0.839   0.118   0.150   0.732
B V  1G3IA                273    0.126  19.428  -0.419   0.118   0.150   0.732
B S  1G3IA                274    0.222  26.065  -1.230   0.113   0.087   0.800
E R  1G3IA                275    0.435  99.523  -0.821   0.113   0.043   0.844
E E  1G3IA                276    0.548  95.701  -0.671   0.268   0.043   0.689
E G  1G3IA                277    0.405  31.889  -1.039   0.354   0.048   0.598
B V  1G3IA                278    0.236  36.304   0.150   0.455   0.046   0.498
B Q  1G3IA                279    0.249  44.436  -0.025   0.717   0.014   0.269
E R  1G3IA                280    0.448 102.546   0.989   0.717   0.014   0.269
E D  1G3IA                281    0.425  61.300  -0.219   0.717   0.014   0.269
B L  1G3IA                282    0.094  17.248   0.599   0.717   0.014   0.269
B L  1G3IA                283    0.107  19.592   0.564   0.717   0.014   0.269
E P  1G3IA                284    0.561  79.535   1.273   0.622   0.015   0.363
E L  1G3IA                285    0.264  48.357  -0.466   0.522   0.016   0.462
B V  1G3IA                286    0.108  16.661   0.569   0.354   0.048   0.598
E E  1G3IA                287    0.488  85.271  -0.263   0.191   0.086   0.723
E G  1G3IA                288    0.323  25.396  -0.983   0.118   0.150   0.732
E S  1G3IA                289    0.423  49.540  -0.697   0.056   0.142   0.802
E T  1G3IA                290    0.500  69.364  -0.892   0.064   0.216   0.721
B V  1G3IA                291    0.180  27.712   0.552   0.064   0.216   0.721
E S  1G3IA                292    0.433  50.748  -0.371   0.056   0.142   0.802
E T  1G3IA                293    0.337  46.714  -0.222   0.056   0.142   0.802
E K  1G3IA                294    0.551 113.300  -1.385   0.056   0.142   0.802
E H  1G3IA                295    0.344  62.628  -0.271   0.020   0.205   0.775
E G  1G3IA                296    0.384  30.221  -1.760   0.021   0.279   0.699
E M  1G3IA                297    0.473  94.627  -1.030   0.021   0.279   0.699
B V  1G3IA                298    0.086  13.218   0.039   0.020   0.205   0.775
E K  1G3IA                299    0.456  93.758  -0.055   0.020   0.205   0.775
B T  1G3IA                300    0.256  35.479  -0.987   0.018   0.088   0.893
E D  1G3IA                301    0.507  73.116  -1.068   0.018   0.047   0.935
B H  1G3IA                302    0.218  39.600  -0.754   0.018   0.047   0.935
B I  1G3IA                303    0.096  17.704  -1.699   0.021   0.279   0.699
B L  1G3IA                304    0.115  20.983  -0.942   0.021   0.451   0.528
B F  1G3IA                305    0.056  11.199  -0.425   0.022   0.552   0.426
B I  1G3IA                306    0.052   9.528  -0.721   0.022   0.552   0.426
B A  1G3IA                307    0.059   6.469  -1.350   0.021   0.451   0.528
B S  1G3IA                308    0.121  14.228  -1.945   0.021   0.279   0.699
B G  1G3IA                309    0.149  11.687  -1.989   0.020   0.205   0.775
B A  1G3IA                310    0.145  15.979  -0.544   0.056   0.142   0.802
B F  1G3IA                311    0.268  53.828  -0.441   0.052   0.084   0.864
E Q  1G3IA                312    0.390  69.565  -0.916   0.053   0.043   0.903
E V  1G3IA                313    0.409  62.848  -0.628   0.113   0.043   0.844
B A  1G3IA                314    0.156  17.224  -0.611   0.113   0.087   0.800
E R  1G3IA                315    0.448 102.661   0.472   0.199   0.152   0.649
E P  1G3IA                316    0.502  71.220  -0.381   0.199   0.152   0.649
E S  1G3IA                317    0.366  42.872  -0.890   0.191   0.086   0.723
E D  1G3IA                318    0.337  48.547  -1.096   0.191   0.086   0.723
B L  1G3IA                319    0.142  25.909  -0.129   0.191   0.086   0.723
B I  1G3IA                320    0.097  18.037   0.749   0.386   0.097   0.517
E P  1G3IA                321    0.300  42.556  -0.443   0.386   0.097   0.517
B E  1G3IA                322    0.286  49.982  -0.375   0.386   0.097   0.517
B L  1G3IA                323    0.155  28.381  -1.078   0.307   0.165   0.527
B Q  1G3IA                324    0.233  41.525  -0.492   0.216   0.235   0.548
B G  1G3IA                325    0.233  18.369  -2.285   0.118   0.150   0.732
E R  1G3IA                326    0.280  64.166  -0.390   0.118   0.150   0.732
B L  1G3IA                327    0.148  27.044  -0.238   0.113   0.087   0.800
B P  1G3IA                328    0.261  37.022  -0.314   0.113   0.087   0.800
B I  1G3IA                329    0.148  27.325  -0.582   0.125   0.227   0.648
B R  1G3IA                330    0.193  44.197   0.051   0.159   0.516   0.325
B V  1G3IA                331    0.103  15.770  -0.203   0.079   0.592   0.329
E E  1G3IA                332    0.354  61.861   0.585   0.074   0.484   0.442
B L  1G3IA                333    0.121  22.210   0.342   0.020   0.205   0.775
E T  1G3IA                334    0.563  78.102   1.014   0.018   0.088   0.893
E A  1G3IA                335    0.586  64.566   1.138   0.053   0.043   0.903
B L  1G3IA                336    0.132  24.188   0.689   0.018   0.019   0.964
E S  1G3IA                337    0.469  54.932   1.395   0.005   0.015   0.979
E A  1G3IA                338    0.475  52.312   1.549   0.923   0.002   0.076
E A  1G3IA                339    0.757  83.410   1.402   0.923   0.002   0.076
B D  1G3IA                340    0.181  26.082   1.279   0.970   0.001   0.030
B F  1G3IA                341    0.046   9.232   0.261   0.970   0.001   0.030
E E  1G3IA                342    0.275  47.973   1.822   0.970   0.001   0.030
E R  1G3IA                343    0.479 109.645   1.966   0.923   0.002   0.076
B I  1G3IA                344    0.044   8.066   0.775   0.923   0.002   0.076
B L  1G3IA                345    0.076  13.971   0.032   0.782   0.003   0.216
E T  1G3IA                346    0.410  56.839   0.108   0.502   0.002   0.495
E E  1G3IA                347    0.421  73.601   0.443   0.176   0.004   0.820
B P  1G3IA                348    0.223  31.601   0.298   0.109   0.005   0.886
E H  1G3IA                349    0.621 113.014  -0.111   0.176   0.004   0.820
B A  1G3IA                350    0.327  36.035  -1.741   0.321   0.003   0.675
B S  1G3IA                351    0.246  28.808  -1.376   0.694   0.003   0.303
B L  1G3IA                352    0.118  21.533  -0.052   0.923   0.002   0.076
B T  1G3IA                353    0.174  24.175   0.045   0.970   0.001   0.030
E E  1G3IA                354    0.425  74.300   1.114   0.970   0.001   0.030
B Q  1G3IA                355    0.177  31.559   0.355   0.970   0.001   0.030
B Y  1G3IA                356    0.070  14.874   0.840   0.970   0.001   0.030
E K  1G3IA                357    0.351  72.242   1.421   0.970   0.001   0.030
E A  1G3IA                358    0.408  45.017   1.039   0.970   0.001   0.030
B L  1G3IA                359    0.078  14.227  -0.370   0.923   0.002   0.076
B M  1G3IA                360    0.116  23.252   0.356   0.858   0.002   0.139
E A  1G3IA                361    0.621  68.445   0.692   0.717   0.014   0.269
B T  1G3IA                362    0.235  32.525  -0.316   0.430   0.016   0.555
E E  1G3IA                363    0.471  82.354  -0.340   0.113   0.043   0.844
E G  1G3IA                364    0.661  52.029   0.154   0.005   0.015   0.979
B V  1G3IA                365    0.078  12.019   0.627   0.021   0.279   0.699
E N  1G3IA                366    0.513  75.045   1.574   0.018   0.846   0.136
B I  1G3IA                367    0.061  11.303   0.978   0.001   0.900   0.099
E A  1G3IA                368    0.503  55.387   1.693   0.001   0.900   0.099
B F  1G3IA                369    0.068  13.688   0.655   0.004   0.616   0.381
E T  1G3IA                370    0.325  45.147   1.285   0.018   0.088   0.893
E T  1G3IA                371    0.673  93.373   0.830   0.858   0.002   0.139
E D  1G3IA                372    0.585  84.313   1.676   0.923   0.002   0.076
B A  1G3IA                373    0.025   2.799   0.698   0.970   0.001   0.030
B V  1G3IA                374    0.043   6.532   0.461   0.970   0.001   0.030
E K  1G3IA                375    0.416  85.571   2.086   0.970   0.001   0.030
E K  1G3IA                376    0.307  63.170   2.072   0.970   0.001   0.030
B I  1G3IA                377    0.032   5.865   0.896   0.970   0.001   0.030
B A  1G3IA                378    0.021   2.292   1.142   0.988   0.000   0.012
E E  1G3IA                379    0.477  83.349   2.167   0.970   0.001   0.030
B A  1G3IA                380    0.211  23.241   2.020   0.970   0.001   0.030
B A  1G3IA                381    0.018   1.984   0.790   0.970   0.001   0.030
B F  1G3IA                382    0.164  32.955   0.506   0.970   0.001   0.030
E R  1G3IA                383    0.592 135.568   1.617   0.923   0.002   0.076
B V  1G3IA                384    0.224  34.413   0.149   0.782   0.003   0.216
E N  1G3IA                385    0.372  54.490  -0.915   0.502   0.002   0.495
E E  1G3IA                386    0.489  85.463  -0.616   0.406   0.004   0.590
E K  1G3IA                387    0.529 108.815  -0.366   0.430   0.016   0.555
E T  1G3IA                388    0.343  47.505  -1.074   0.339   0.016   0.645
E E  1G3IA                389    0.448  78.301  -0.205   0.268   0.043   0.689
E N  1G3IA                390    0.400  58.501  -0.663   0.268   0.043   0.689
B I  1G3IA                391    0.090  16.650   0.101   0.354   0.048   0.598
B G  1G3IA                392    0.114   8.972  -1.963   0.522   0.016   0.462
B A  1G3IA                393    0.070   7.692  -1.633   0.622   0.015   0.363
E R  1G3IA                394    0.235  53.815   0.182   0.717   0.014   0.269
B R  1G3IA                395    0.138  31.533  -0.200   0.879   0.010   0.111
B L  1G3IA                396    0.033   5.969   0.057   0.923   0.002   0.076
B H  1G3IA                397    0.202  36.762   0.476   0.970   0.001   0.030
B T  1G3IA                398    0.111  15.465   0.282   0.970   0.001   0.030
B V  1G3IA                399    0.044   6.763   0.325   0.970   0.001   0.030
B M  1G3IA                400    0.034   6.843   0.271   0.988   0.000   0.012
B E  1G3IA                401    0.106  18.571  -0.004   0.988   0.000   0.012
E R  1G3IA                402    0.361  82.600   1.423   0.970   0.001   0.030
B L  1G3IA                403    0.042   7.727   0.492   0.970   0.001   0.030
B M  1G3IA                404    0.048   9.625  -0.065   0.923   0.002   0.076
B D  1G3IA                405    0.183  26.399  -0.225   0.923   0.002   0.076
B K  1G3IA                406    0.269  55.333  -0.272   0.858   0.002   0.139
B I  1G3IA                407    0.038   6.993   0.051   0.802   0.014   0.185
B S  1G3IA                408    0.106  12.458  -0.902   0.561   0.047   0.393
B F  1G3IA                409    0.168  33.617  -0.498   0.307   0.165   0.527
E S  1G3IA                410    0.357  41.794   0.200   0.199   0.152   0.649
B A  1G3IA                411    0.046   5.080   0.499   0.058   0.017   0.925
E S  1G3IA                412    0.332  38.887  -0.120   0.018   0.019   0.964
E D  1G3IA                413    0.665  95.870  -0.614   0.016   0.005   0.979
E M  1G3IA                414    0.458  91.726  -0.001   0.018   0.019   0.964
E N  1G3IA                415    0.725 106.111   0.394   0.018   0.047   0.935
E G  1G3IA                416    0.460  36.202  -0.207   0.005   0.045   0.951
E Q  1G3IA                417    0.428  76.459   1.192   0.005   0.262   0.733
E T  1G3IA                418    0.426  59.128   1.957   0.001   0.900   0.099
B V  1G3IA                419    0.055   8.438   1.459   0.001   0.959   0.040
E N  1G3IA                420    0.312  45.706   1.872   0.001   0.959   0.040
B I  1G3IA                421    0.045   8.233   0.898   0.001   0.900   0.099
B D  1G3IA                422    0.212  30.607   1.214   0.023   0.655   0.322
E A  1G3IA                423    0.390  43.011   1.333   0.660   0.049   0.291
E A  1G3IA                424    0.677  74.594   1.483   0.802   0.014   0.185
B Y  1G3IA                425    0.154  32.931   1.604   0.751   0.050   0.199
B V  1G3IA                426    0.039   5.933  -0.177   0.701   0.107   0.192
E A  1G3IA                427    0.554  61.062   1.694   0.561   0.047   0.393
E D  1G3IA                428    0.685  98.766   1.201   0.339   0.016   0.645
E A  1G3IA                429    0.599  66.010   0.796   0.257   0.016   0.727
E L  1G3IA                430    0.300  54.948  -0.012   0.257   0.016   0.727
E G  1G3IA                431    0.554  43.631   1.203   0.339   0.016   0.645
E E  1G3IA                432    0.569  99.457   1.028   0.455   0.046   0.498
B V  1G3IA                433    0.155  23.747   0.488   0.605   0.105   0.290
B V  1G3IA                434    0.187  28.773   0.001   0.538   0.173   0.289
E E  1G3IA                435    0.599 104.628   1.279   0.538   0.173   0.289
E N  1G3IA                436    0.414  60.654   0.724   0.278   0.093   0.628
E E  1G3IA                437    0.572 100.016   1.291   0.181   0.016   0.803
E D  1G3IA                438    0.756 108.954   0.515   0.115   0.016   0.868
B L  1G3IA                439    0.180  32.958   0.996   0.115   0.016   0.868
E S  1G3IA                440    0.434  50.888   0.491   0.113   0.087   0.800
E R  1G3IA                441    0.504 115.439   1.182   0.064   0.216   0.721
E F  1G3IA                442    0.325  65.207   1.029   0.125   0.227   0.648
B I  1G3IA                443    0.228  42.236   0.558   0.118   0.150   0.732
E L  1G3IA                444    0.467  85.416  -1.140   0.003   0.003   0.994

Explain the output.  Go back.
```

---
